# Interrelationships of Sleep Quality, Obesity Severity, and Clinical Headache Features among Women with Comorbid Migraine and Obesity

**DOI:** 10.3390/jcm12051742

**Published:** 2023-02-22

**Authors:** Leah M. Schumacher, Samantha G. Farris, J. Graham Thomas, Richard B. Lipton, Jelena Pavlovic, Angeliki Vgontzas, Dale S. Bond

**Affiliations:** 1 Department of Kinesiology, College of Public Health, Temple University, 1800 N. Broad St., Philadelphia, PA 19121, USA; 2 Department of Psychology, Rutgers, The State University of New Jersey, 53 Avenue East, 211 Tillett Hall, Piscataway, NJ 08854, USA; 3 Department of Psychiatry and Human Behavior, Alpert Medical School, Brown University, Providence, RI 02903, USA; 4 Weight Control and Diabetes Research Center, The Miriam Hospital, 196 Richmond Street, Providence, RI 02903, USA; 5 Department of Neurology and the Montefiore Headache Center, Albert Einstein College of Medicine, 1250 Waters Pl #8, The Bronx, NY 10461, USA; 6 Department of Neurology and the Montefiore Headache Center, Montefiore Medical Center, 1250 Waters Pl #8, The Bronx, NY 10461, USA; 7 Department of Neurology, Brigham and Women’s Hospital, Harvard Medical School, 60 Fenwood Rd 1st Floor, Boston, MA 02115, USA; 8 Departments of Surgery and Research, Hartford Hospital, 80 Seymour St., Hartford, CT 06102, USA

**Keywords:** migraine, sleep, obesity, women’s health

## Abstract

Obesity and migraine are often comorbid. Poor sleep quality is also common among individuals with migraine and may be influenced by comorbidities such as obesity. However, understanding of migraine’s relationship with sleep and the potential exacerbating effect of obesity remains limited. This study evaluated the associations of migraine characteristics and clinical features with sleep quality among women with comorbid migraine and overweight/obesity and assessed the interplay between obesity severity and migraine characteristics/clinical features in relation to sleep quality. Women seeking treatment for migraine and obesity (*n* = 127; NCT01197196) completed a validated questionnaire assessing sleep quality (Pittsburgh Sleep Quality Index-PSQI). Migraine headache characteristics and clinical features were assessed using smartphone-based daily diaries. Weight was measured in-clinic, and several potential confounders were assessed using rigorous methods. Nearly 70% of participants endorsed poor sleep quality. Greater monthly migraine days and the presence of phonophobia related to poorer sleep quality, and specifically poorer sleep efficiency, controlling for confounders. Obesity severity was neither independently associated nor interacted with migraine characteristics/features to predict sleep quality. Poor sleep quality is common among women with comorbid migraine and overweight/obesity, although obesity severity does not appear to uniquely relate to or exacerbate the association between migraine and sleep in this population. Results can guide research on mechanisms of the migraine–sleep link and inform clinical care.

## 1. Introduction

Migraine is a neurological condition characterized by recurrent moderate-to-severe headaches with accompanying sensory, autonomic, and affective features [1]. Migraine affects approximately 12% of the population [2] and is more prevalent and often experienced as more chronic and severe among individuals with obesity, especially women [3,4,5,6]. Poor sleep quality is common among individuals with migraine [7,8,9,10]. The relationships between sleep and migraine are complex, bidirectional, and multifactorial; migraine can interfere with sleep and poor sleep can trigger a migraine attack and exacerbate pain sensitivity [10,11]. The association of poor sleep quality and migraine may be explained by common pathophysiological mechanisms, shared comorbidities, or both [10].

One comorbidity that may influence the sleep–migraine link but has received limited attention is obesity. Individuals with comorbid migraine and overweight/obesity may be especially prone to poor sleep quality [12]. For example, both excess weight and migraine headache symptoms have potential to disturb sleep (e.g., due to pain, breathing-related difficulties) and to affect aspects of sleep including sleep quality and efficiency. Both migraine and obesity are also associated with increased fatigue [13,14,15], which could affect behavioral sleep patterns (e.g., napping [16], more time spent in bed). When migraine and overweight/obesity are experienced together, the impact on sleep may be heightened. However, limited research has examined sleep quality in the context of comorbid migraine and overweight/obesity.

Additionally, while several sensory and autonomic features associated with migraine may affect sleep among this patient population, limited research has investigated the association between these symptoms and sleep. For example, allodynia (i.e., abnormal pain in response to tactile stimuli, such as light touch or wearing jewelry), photophobia (i.e., hypersensitivity to light), and phonophobia (i.e., hypersensitivity to sound) may all make it more difficult for individuals to fall or stay asleep due to heightened responses to normative tactile stimuli and ambient light and sound [17]. Likewise, the physical discomfort associated with migraine-related nausea may interfere with sleep [18]. Improved understanding of migraine characteristics and clinical features that relate to impaired sleep quality is critical to guide research on underlying mechanisms of the migraine–sleep link and to inform clinical intervention (e.g., identify who may be at greatest risk for poor sleep and might benefit from additional sleep-related intervention) [19].

A pragmatic strategy for learning more about sleep quality in the context of comorbid migraine and overweight/obesity is through use of secondary analysis of existing clinical trial data. The Women’s Health and Migraine (WHAM) study [20] was a randomized trial comparing behavioral and educational interventions to reduce weight and headache frequency and severity. All participants had overweight/obesity, met diagnostic criteria for migraine, and completed validated measures of sleep and several potential confounders during the pretreatment baseline phase of the WHAM trial. The current study aimed to leverage the pretreatment data from the WHAM trial to evaluate sleep quality and its associations with migraine characteristics, cardinal clinical features, and obesity severity. The potential impact of obesity severity on the associations between migraine and sleep was also evaluated.

The primary aims were to: (1) assess the relative frequency of poor sleep quality among a sample of women with comorbid migraine and overweight/obesity; (2) evaluate the associations of obesity severity and of migraine characteristics (monthly migraine days [MMD], pain intensity, attack duration), cardinal clinical features (nausea, photophobia, and phonophobia), and allodynia with overall sleep quality and specific dimensions of sleep quality, when controlling for potential confounders; and (3) assess the interplay between obesity severity and migraine characteristics/clinical features in relation to sleep quality. We hypothesized that: (1) poor sleep quality would be common in this population, (2) more frequent or severe migraine characteristics and clinical features, as well as greater obesity severity, would relate to poorer sleep quality, and (3) obesity severity would moderate the association of migraine characteristics/clinical features with sleep quality, such that more frequent or severe migraine characteristics/clinical features would be most strongly related to poorer sleep quality among those with more severe obesity.

## 2. Materials and Methods

The current study involved a secondary exploratory analysis of data from a randomized clinical trial that compared behavioral weight loss to migraine education for decreasing headache among women with comorbid migraine and obesity (the WHAM study) [20].

Participants were recruited from the community and neurological clinical settings from November 2012 to March 2016. Eligibility required identifying as a woman, being 18–50 years old, having both overweight/obesity (body mass index [BMI]: 25.0–49.9 kg/m^2^) and neurologist-confirmed migraine (see below), and experiencing ≥3 migraine attacks and 4 to 20 migraine headache days during each of the past 3 months. For the parent trial, the decision was made to focus only on women because the association between migraine and obesity is strongest in women of reproductive age [3]. Exclusion criteria included headache disorder other than migraine or migraine with tension-type; current participation in a weight loss program or use of prescription weight loss medication; previous bariatric surgery; ≥5% weight loss in the past 6 months; current pregnancy, breastfeeding, or plans to become pregnant during the study period; cancer diagnosis in the past year; and presence of another condition that the study team believed may preclude adherence to the protocol (e.g., plans to move out of the area, severe psychiatric problem). The WHAM protocol details, including full inclusion/exclusion criteria, were previously published [20]. The study took place at the Weight Control and Diabetes Research Center of The Miriam Hospital and was approved by The Miriam Hospital’s IRB. Informed consent was obtained from all participants involved in the study. The parent study was registered at clinicaltrials.gov (NCT01197196); the secondary exploratory analyses reported here were not preregistered. All data utilized in the present study were collected as part of the baseline assessment for the parent trial, prior to any clinical intervention. Participants did not receive monetary compensation for completing the baseline assessment.

### 2.1. Migraine Diagnosis, BMI, and Medical History

Migraine diagnosis was confirmed by a neurologist using International Classification for Headache Disorders third edition criteria [21]. Weight and height were measured via a digital scale and stadiometer, respectively; a body mass index (BMI) of ≥25.0 kg/m^2^ indicated the presence of overweight or obesity. Participants completed a health history questionnaire, which assessed lifetime diagnosis of obstructive sleep apnea (OSA; Yes/No) and current continuous positive airway pressure (CPAP) machine use.

### 2.2. Sleep Quality 

The Pittsburgh Sleep Quality Index (PSQI) [22] is a 19-item self-report assessment of past 30-day sleep quality. A global score can be derived (possible range 0 to 21), with higher scores indicating worse overall sleep quality. A score of >5 denotes poor sleep quality and a score of ≤5 indicates good sleep quality [22]. In addition to producing a global score, the PSQI allows for evaluation of distinct sleep domains. While the PSQI can yield scores for seven sleep domains (sleep duration, habitual sleep efficiency, sleep latency, subjective sleep quality, frequency of sleep medication use, sleep disturbances, and daytime dysfunction) [22], a previously validated three component scoring system can also be used for parsimony and to limit the number of statistical tests conducted while still obtaining meaningful information about different aspects of sleep quality [23,24]. This alternate scoring system combines items from each of the seven potential sleep domain scores into the following three components: sleep efficiency (i.e., sleep duration + habitual sleep efficiency items), perceived sleep quality (i.e., sleep latency, subjective sleep quality, + frequency of sleep medication use items), and daily disturbances (i.e., sleep disturbances + daytime dysfunction items) [23,24]. Given our interest in assessing the associations among sleep quality and multiple migraine characteristics/clinical features, we utilized the three component scoring method to minimize risk of type I error.

### 2.3. Migraine Characteristics, Clinical Features, and Allodynia

Migraine characteristics and clinical features were assessed via daily diary. Participants received a smartphone equipped with a diary application to report on migraine headache activity for 28 days, including migraine headache occurrence each day (Yes/No; several follow-up items differentiated migraine headaches from non-migraine headaches [e.g., unilateral vs. bilateral pain, pulsating/throbbing vs. pressing/tightening pain]), maximum pain intensity (0–10), attack duration (hours), and presence of the following clinical features: nausea, photophobia, and phonophobia. The following variables were derived from the diary responses: MMD, average maximum pain intensity, average attack duration, and percentage of episodes at which each clinical feature was endorsed. Diary responses were checked daily for completeness and participants were contacted by phone to obtain missing data. In addition, the severity of cutaneous allodynia symptoms was assessed using the Allodynia Symptom Checklist [25], a 12 item self-report measure that yields a total severity score (possible range 0 to 24), with higher values reflecting greater symptomatology.

### 2.4. Potential Confounders 

The Center for Epidemiological Studies-Depression (CES-D) [26] scale was used to assess depression, with scores ≥16 indicting clinically meaningful depressive symptoms, and the Generalized Anxiety Disorder Questionnaire (GAD-7) [27] was used to assess anxiety, with scores ≥10 indicating clinically elevated anxiety symptoms. The Perceived Stress Scale [28] was used to assess stress levels. Average daily minutes of moderate-to-vigorous physical activity was mesaured with a SenseWear Mini Armband monitor (BodyMedia Inc., Pittsburgh, PA, USA), which participants were asked to wear over the upper left triceps muscle during all waking hours for 7 consecutive days. This multi-sensor monitor integrates data from a triaxial acceleromter, physiologic metrics from several sensors (e.g., galvanic skin response), and participant demographic information (e.g., sex, body weight) to estimate the intensity of activities using proprietary software (SenseWear Professional Software, version 7.0). The SenseWear monitor has been shown to provide estimates of time spent in activity of various intensities similar to other monitors [29,30]. A metabolic equivalents value of ≥3 was used to classify daily minutes of moderate-to-vigorous physical activity [31]. Caffeine intake and alcohol intake was assessed via three, nonconseutive (two weekday and one weekend), mulitple-pass, 24-h diet recalls. Each recall was conducted over the phone by a trained interviewer using Nutrition Data Systems for Research (Version 2013, Nutrition Coordinating Center (NCC), University of Minnesota, Minneapolis, MN) [32]. Data from the weekdays (Monday-Friday) and weekend days (Saturday and Sunday) were weighted to create average daily caffeine (in mg) and average daily alcohol (in g) intake variables [33].

### 2.5. Analytic Plan

**Figure 1:**
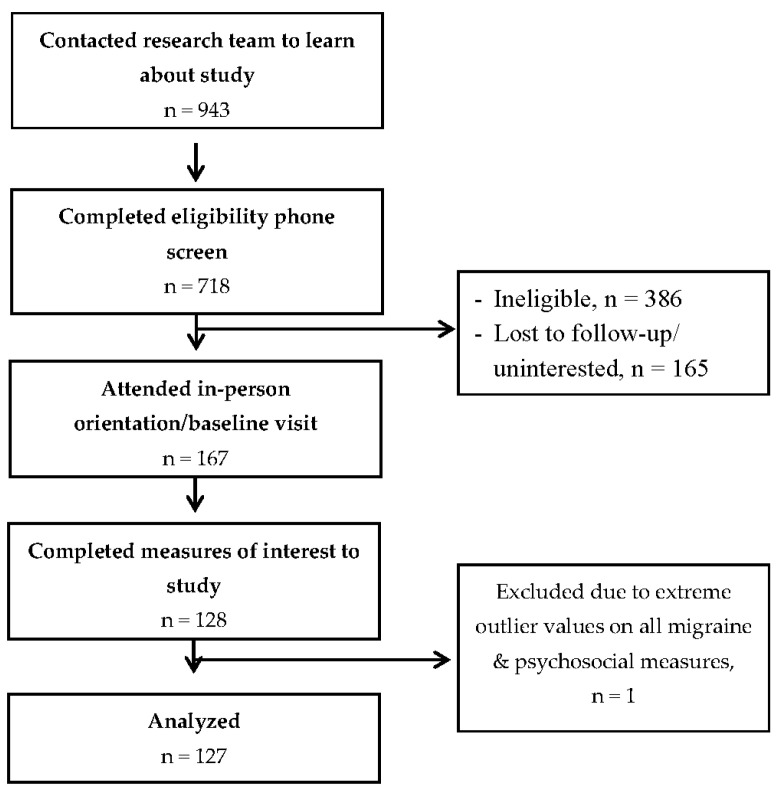
Study flow.

Data were analyzed using SPSS version 25. The threshold for statistical significance was set at 0.05 with two-tailed tests. Data were screening for missingness, outliers, and normality. Data from one participant were excluded due to extreme outlier values reported across measures that led to violations of model assumptions. One participant was missing data on diary-assessed migraine clinical features (nausea, photophobia, phonophobia) and was excluded from that subset of analyses. Two cases were excluded from analyses pertaining to migraine attack duration due to outlier values. Thus, data from a total of 127 participants were analyzed in most models, with 126 included in analyses pertaining to diary-assessed clinical features and 125 included in analyses of migraine attack duration. See Figure 1 for an outline of participant flow.

For outcome variables, PSQI global scores and the PSQI daily disturbance scores were normally distributed. PSQI sleep efficiency and sleep quality scores were non-normally distributed (i.e., skewness and/or kurtosis values more than twice its standard error). Data transformations (square root for efficiency and natural log for sleep quality) were thus performed to achieve more normal distributions for analyses. For migraine variables, MMD, attack duration, and allodynia were positively skewed and were also transformed. However, as the same pattern of results was observed when conducting analyses with untransformed and transformed independent variables; values from models with untransformed independent variables are reported to enhance interpretability.

The distributions of phonophobia and photophobia scores were highly skewed, with modes at the minimum (i.e., 58.3% of participants reported phonophobia at no episodes) or maximum (i.e., 60.6% of participants reported photophobia at every episode) values, respectively. We thus categorized participants as experiencing phonophobia at no (0%) or any (1–100%) episodes and as experiencing photophobia at no/some (0–99%) or all (100%) episodes. The distribution for nausea was also non-normal, exhibiting a bimodal distribution with 17.9% of participants reporting nausea at no episodes and 16.4% reporting nausea at every episode. We thus categorized participants as low (0–33.3%), medium (33.4–66.7%) or high (66.8–100%) for nausea.

Variables were descriptively characterized with means and standard deviations and medians and the interquartile range (continuous variables) or with frequencies and percentages (categorical and ordinal variables). Associations of each migraine characteristic/clinical feature with sleep quality (global score and each of the three domains) and its interaction with BMI were assessed using separate hierarchical linear regression models. The migraine variable of interest (MMD, pain intensity, attack duration, allodynia, nausea, phonophobia, or photophobia), BMI, and potential confounders (anxiety, depression, stress, average daily moderate-to-vigorous physical activity, average daily caffeine intake, average daily alcohol intake) were entered in the first step. The interaction of BMI and the migraine variable was then entered in the second step. BMI was centered, and migraine variables were centered if continuous or dummy-coded if categorical or ordinal (reference group = no or minimal symptoms). The sample size was determined through a power analysis for the main trial’s primary outcomes [20].

## 3. Results

### 3.1. Participant Flow and Descriptive Characteristics

Table 1 displays participant characteristics for the overall sample and for participants with poor vs. good sleep, as classified by the PSQI and reported below. Participants had a mean age of approximately 38 years, and a majority identified as White and non-Hispanic. Approximately 61% were married or living with a partner, and approximately 61% had a college or graduate degree. Approximately 6% (*n* = 8) endorsed lifetime diagnosis of OSA, with half of those individuals endorsing current CPAP use. Age, race, ethnicity, educational attainment (as categorized in Table 1), and marital status (as categorized in Table 1) did not relate to overall sleep quality or BMI (*p*’s > 0.10). However, participants who reported lifetime OSA had higher average BMI’s (*M* = 42.2, *SD* = 6.2) and poorer overall sleep quality (*M* = 11.1, *SD* = 3.9) than those without OSA (BMI: *M* = 35.0, *SD* = 6.4; F = 9.34, *p* = 0.003; PSQI global score: *M* = 7.7, *SD* = 3.6, F = 6.78, *p* = 0.010). 

Table 2 and Table 3 show descriptive statistics for the full sample (Table 2) and for those with poor vs. good sleep (Table 3). Notably, a majority (69.3%) of participants were classified as having poor sleep based on their PSQI score, while 30.7% were classified as having good sleep. The average BMI was approximately 35 kg/m^2^, indicating class II obesity. Participants reported an average of approximately 8 MMD, a mean maximum pain intensity of 6, a mean attack duration of 18 h, and a mean allodynia score of approximately 5, indicating mild allodynia. 

### 3.2. Migraine Characteristics/Clinical Features and BMI in Relation to Sleep Quality

Similar patterns of results were observed across all regression models. Table 4 thus presents an example full model output for one of the hierarchical regression models predicting overall sleep quality. As shown, the first step in all models significantly predicted sleep quality, with several confounders (e.g., depression) emerging as independently related to sleep quality. BMI was not related to sleep quality in any model, and the inclusion of the interaction between BMI and the migraine characteristic/clinical feature (e.g., MMD) in step two did not improve prediction in any model. Given this consistent pattern of findings, Table 5 presents a summary of results from the other hierarchical linear regression models, displaying only b values for the association between each migraine characteristic/clinical feature and the sleep outcome of interest, when adjusting for BMI and confounders in step one.

**Figure 2:**
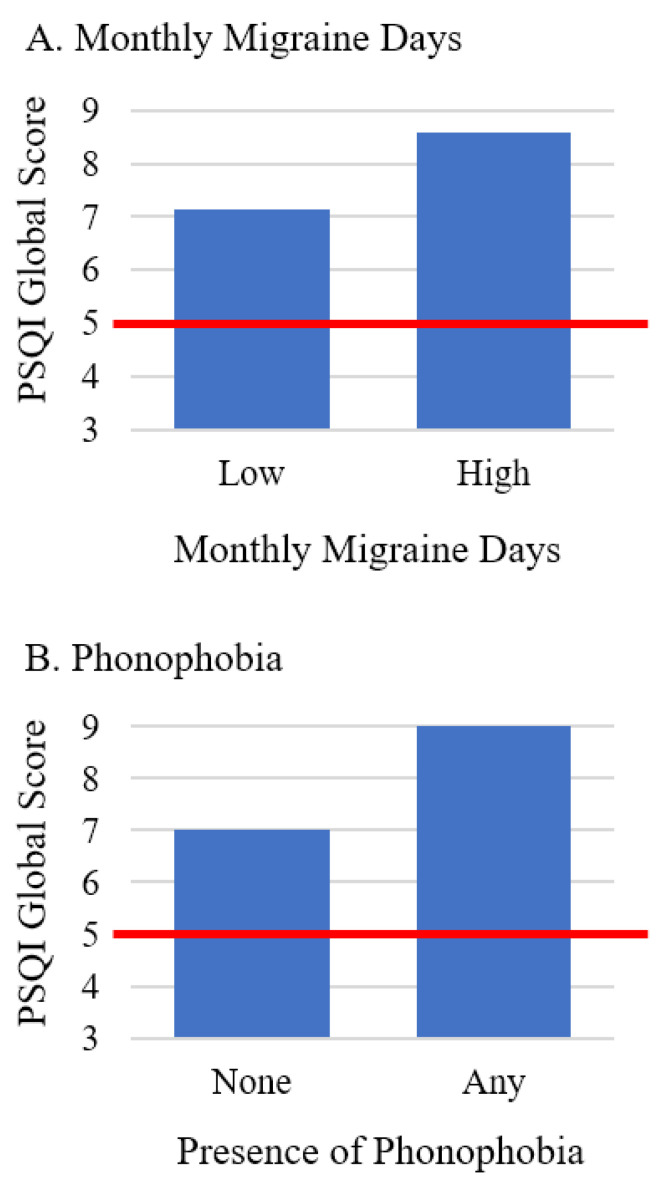
Differences in overall sleep quality by monthly migraine days (**A**) and presence of phonophobia (**B**), after controlling for body mass index and potential confounders. Note. The red line indicates the cutoff for poor sleep quality, with higher PSQI scores indicating worse sleep quality. Monthly migraine days were dichotomized for illustrative purposes. Participants were categorized as having phonophobia at none vs. any migraine episodes. Abbreviations: PSQI = Pittsburgh Sleep Quality Index.

As shown in Table 5, MMD and phonophobia were independently related to sleep quality when controlling for BMI and potential confounders (*p*’s < 0.05). Figure 2 displays differences in overall sleep quality based on MMD and phonophobia. Regarding specific dimensions of sleep quality, sleep efficiency was the only dimension of sleep quality related to migraine characteristics/clinical features, with MMD, nausea, and phonophobia all relating to sleep efficiency (*p*’s < 0.05). 

## 4. Discussion

This study examined sleep quality and its association with several migraine characteristics/features and obesity severity among a treatment-seeking sample of women with comorbid migraine and overweight or obesity. Our results suggest that poor sleep is common in this population—a population that continues to increase as obesity rates rise. We found that nearly 70% of our sample had poor sleep quality based on a validated questionnaire. This high rate of poor sleep quality is consistent with the broader literature on sleep quality among individuals with migraine [7,8,9,10,23] and is higher than rates observed in some studies with migraine and headache patients [7,23]. The higher rates of poor sleep quality in the present sample could be due to all individuals having overweight or obesity, which has been shown to itself relate to poorer sleep quality [34,35].

Although past research shows presence of overweight/obesity relates to poorer sleep quality in the general population [34,35], obesity severity, as measured by BMI, was not related to sleep quality in our sample. These unexpected findings could be due to the restricted range in BMI, the limited ability of the PSQI to detect sleep problems most relevant to obesity (e.g., OSA-related), or the modest rate of OSA in this sample. In partial support of a role for OSA, participants who endorsed OSA reported both poorer overall sleep quality and more severe obesity than those without OSA. While caution is needed when interpreting these findings given that only eight participants endorsed OSA, it may be that obesity severity is most relevant to sleep-related breathing disorders, such as OSA and its associated symptoms among individuals with migraine. Obesity severity may be less relevant to other aspects of sleep quality, which may be affected among many individuals across the overweight/obesity spectrum. It is also possible that the relationships between migraine, obesity, and sleep quality differ or are moderated by OSA; we were underpowered to explore such relationships. Given that obesity is a major risk factor for OSA [36] and that OSA may impact migraine headache symptoms [11], additional research on the associations of OSA, obesity severity, and migraine is needed. Inclusion of both men and women (vs. just women) in these studies will be important, as OSA is more prevalent among men [37].

When evaluating overall sleep quality, we also found that poorer overall sleep was related to several specific migraine characteristics/features, namely greater MMD and the presence of phonophobia. These findings are consistent with some prior research [7,8,38]. Although we cannot determine directionality from this cross-sectional study, given the bidirectional associations between migraine and sleep [10,11] it may be that those with poorer sleep are prone to having more frequent migraine attacks and/or that more frequent migraine attacks negatively affect sleep. Such associations could be due to both behavioral and biological factors. For example, more frequent discomfort from regular headaches could make it difficult for individuals to fall and stay asleep, or dysfunction in brainstem networks involved in switching between sleep stages (e.g., networks in the hypothalamus) in patients with migraine could negatively affect important aspects of sleep such as amount of time in slow wave sleep and sleep–wake transitions [11]. For the phonophobia findings, it is possible that sensitivity to sound (but not light) may uniquely interfere with sleep as it may be easier to escape light in a dark room than it is to escape sound. Again, physiological abnormalities that lead to sensory hypersensitivity among migraine patients could also affect biological mechanisms involved in sleep regulation [11]. These findings are consistent with the notion that migraine is a disorder of sensory amplification, although additional research is warranted to elucidate mechanisms that link sensory disturbances and poor sleep quality in migraine [39]. Notably, the observed associations between migraine characteristics/features and poor sleep quality were significant above and beyond relevant factors know to affect sleep quality, including mood and stress-related variables, physical activity, and caffeine and alcohol intake. These findings underscore the importance of assessing and attending to sleep complaints in people with migraine, as sleep interventions may not only improve sleep but also migraine frequency and severity [19,40].

When evaluating how specific components of sleep related to migraine characteristics/clinical features to deepen understanding of the associations discussed above, we found that sleep efficiency, which reflects sleep duration and the percentage of the total time in bed that one spends asleep, was the specific dimension of overall sleep quality that related to migraine characteristics/clinical features (i.e., MMD, phonophobia, nausea). These findings add to a mixed literature on migraine and sleep efficiency and duration [23,41,42,43]. Discrepant findings across studies may be due to differences in methodologies (e.g., self-report vs. actigraphy measures of sleep), study designs (e.g., studies assessing day-to-day associations between migraine and sleep vs. studies assessing these associations on average across several weeks), and sample characteristics (e.g., inclusion of individuals across the weight spectrum vs. only those with overweight/obesity, which itself relates to shorter sleep duration and poorer efficiency [44,45,46]). These data help elucidate which migraine characteristics/clinical features relate to different aspects of sleep quality, with potential to inform both mechanistic research and clinical care [40].

The major strengths of the current study include: evaluation of associations of both migraine characteristics and clinical features with sleep quality in a sample of individuals with comorbid migraine and overweight/obesity who are thus at potential elevated risk for poor sleep; use of smartphone-based daily diaries to obtain ecologically valid assessment of migraine characteristics and clinical features; and consideration of numerous confounders, including actigraphy-assessed physical activity and caffeine and alcohol intake as assessed by interviewer-administered recalls. Limitations include inclusion of only women, assessment of sleep quality at a single timepoint using a retrospective questionnaire, no assessment of sleep disorders other than OSA, and reliance on self-report of OSA diagnosis using a health history questionnaire (vs. a more validated OSA screener such as STOP-Bang) [47]. The study also did not assess day-to-day associations between sleep and migraine (i.e., the short-term effects of migraine today on sleep tonight or the effects of sleep tonight on migraine tomorrow), nor did it examine the influence of nocturnal migraine on sleep. Additionally, the sample was less racially and ethnically diverse than the U.S. population. It is critical for future studies assessing the migraine–sleep link to have more representative samples, particularly given significant disparities in migraine prevalence, pain and disability, and care [48,49]. Future studies would also benefit from including men and women; using both subjective measurement of sleep quality and objective measurement of physiological sleep paramaters; using a validated screener such as STOP-Bang or polysomnography to assess OSA; and further exploring optimal approaches for evaluating specific sleep domains when using the PSQI. It would also be advantagoues to apply a more interdisciplinary lens to understanding the relationships among migraine, sleep, and obesity (e.g., evaluating potential physiological abnormalities that contribute to poor sleep in this population, including hypothamalic dyfunction or disruptions in melatonin production [50]) and to consider assessing these associations over time to help tease apart these complex associations (e.g., evaluating whether sleep improves following successful migraine treatment that reduces migraine-related symptoms even if weight remains stable).

## 5. Conclusions

In conclusion, this study shows that poor sleep quality is highly prevalent among women with migraine and overweight/obesity, although obesity severity was not uniquely related to sleep quality and did not moderate the associations between migraine and sleep quality. Greater MMD and the presence of phonophobia, in particular, were related to poor sleep quality, as reflected by both global sleep scores and sleep efficiency scores. Perhaps treating migraine to reduce MMDs and pain sensitization with effective pharmacological and non-pharmacological treatment may improve both migraine and sleep, including among individuals with both migraine and obesity. Results can inform research seeking to elucidate the complex relationship between migraine and sleep. Pending replication, these findings may also assist in identifying sleep phenotypes that contribute to differences in migraine, enable better treatment matching, and inform new targets for tailored, innovative, and effective sleep interventions.

## Figures and Tables

**Table 1:** Participant characteristics.

Characteristic	Full Sample (*n* = 127)	Participants with Poor Sleep (*n* = 88)	Participants with Good Sleep (*n* = 39)
Age (years), *M* (*SD*)	38.3 (8.0)	38.2 (7.7)	38.6 (8.8)
Race, *n* (%)			
Black	16 (12.6%)	14 (15.9%)	2 (5.1%)
White	97 (76.4%)	63 (71.6%)	34 (87.2%)
More than one race	3 (2.4%)	3 (3.4%)	0
Other	11 (8.7%)	8 (9.1%)	3 (7.7%)
Ethnicity, *n* (%)			
Hispanic	23 (18.1%)	17 (19.3%)	6 (15.4)
Not Hispanic	104 (81.9%)	71 (80.7%)	33 (84.6%)
Marital Status, *n* (%)			
Married or living with significant other	77 (60.6%)	49 (55.7%)	28 (71.8%)
Separated or divorced	16 (12.6%)	10 (11.4%)	6 (15.4%)
Never married	32 (25.2%)	27 (30.7%)	5 (12.8%)
Other	2 (1.6%)	2 (2.3%)	0
Educational attainment, *n* (%)			
High school degree or less	12 (9.4%)	8 (9.1%)	4 (10.3%)
Vocational training or some college	38 (30.0%)	24 (27.3%)	14 (35.9%)
College/university degree	52 (40.9%)	37 (42.0%)	15 (38.5%)
Graduate degree	25 (19.7%)	19 (21.	6 (15.4%)
Obstructive sleep apnea			
Lifetime diagnosis	8 (6.3%)	7 (8.0%)	1 (2.6%)
Current use of CPAP machine	4 (3.1%)	3 (3.4%)	1 (2.6%)

Note. Abbreviations: *M* = mean, *SD* = standard deviation, CPAP = continuous positive airway pressure. Descriptive characteristic report on the sample used in most analyses (*n* = 127). A *t*-test comparing age and Chi-square tests comparing race, ethnicity, marital status, and educational attainment between participants with good vs. poor sleep revealed no statistically significant differences (*p*’s > 0.10). Groups could not be compared for lifetime diagnosis of OSA and CPAP use due to small cell size. The sum of percentages for a particular category may exceed 100% due to rounding.

**Table 2:** Descriptive characteristics for variables of interest for full sample.

**Continuous Variables**		
**Variable**	**Mean** **(SD)**	**Median** **(25%, 75%)**
PSQI overall subjective sleep quality score	7.9 (3.7)	8.0 (5.0, 11.0)
PSQI sleep efficiency score	2.2 (1.7)	2.0 (1.0, 3.0)
PSQI subjective sleep quality score	3.1 (2.0)	3.0 (1.0, 5.0)
PSQI daily disturbances score	2.7 (1.0)	3.0 (2.0, 3.0)
BMI (kg/m^2^)	36.1 (6.6)	34.9 (30.3, 40.4)
Monthly migraine days	8.4 (4.6)	7.0 (5.0, 11.0)
Pain intensity (0–10)	5.8 (1.6)	5.8 (4.8, 7.0)
Attack duration (hours)	18.2 (9.8)	16.0 (12.0, 20.9)
Allodynia	5.1 (3.8)	4.0 (2.0, 7.0)
Perceived stress	16.8 (6.6)	17.0 (12.0, 22.0)
Average daily moderate-to-vigorous physical activity (min)	42.9 (32.4)	36.0 (23.9, 53.1)
Average daily caffeine intake (mg)	152.6 (171.2)	116.9 (40.7, 203.3)
Average daily alcohol intake (g)	4.2 (10.8)	0 (0, 0.3)
**Categorical Variables**		
**Variable**	***n* (%)**	
Nausea frequency		
Low (0–33.3% of episodes)	48 (38.1%)	
Medium (33.4–66.7% of episodes)	42 (33.3%)	
High (66.8–100% of episodes)	36 (29.9%)	
Photophobia (present at all episodes)	77 (60.6%)	
Phonophobia (present at any episodes)	53 (41.7%)	
Anxiety (GAD-7 ≥ 10)	31 (24.4%)	
Depression (CES-D ≥ 16)	56 (44.1%)	

Note. Percentages are for valid data. Abbreviations: PSQI = Pittsburgh Sleep Quality Index, BMI = body mass index, GAD = Generalized Anxiety Disorder Questionnaire, CES-D = Center for Epidemiological Studies-Depression, MMD = monthly migraine days, SD = standard deviation, 25% = 25th percentile, 75% = 75th percentile.

**Table 3:** Descriptive characteristics for variables of interest for individuals with poor vs. good sleep.

**Continuous Variables**				
	**Participants with Poor Sleep**		**Participants with Good Sleep**	
**Variable**	**Mean** **(SD)**	**Median** **(25%, 75%)**	**Mean** **(SD)**	**Median** **(25%, 75%)**
PSQI overall subjective sleep quality score	9.9 (2.5)	10.0 (8.0, 12.0)	3.6 (1.3)	4.0 (3.0, 5.0)
PSQI sleep efficiency score	2.9 (1.5)	3.0 (2.0, 4.0)	0.7 (0.7)	1.0 (0, 1.0)
PSQI subjective sleep quality score	3.9 (1.8)	3.0 (3.0, 5.0)	1.1 (0.8)	1.0 (0, 2.0)
PSQI daily disturbances score	3.1 (0.9)	3.0 (3.0, 4.0)	1.9 (0.8)	2.0 (1.0, 2.0)
BMI (kg/m^2^)	36.1 (6.6)	35.4 (30.6, 40.9)	34.0 (6.3)	33.1 (29.7, 37.3)
Monthly migraine days	8.5 (4.9)	8.0 (5.0, 11.0)	8.4 (4.6)	7.0 (5.0, 11.0)
Pain intensity (0–10)	6.1 (1.6)	6.0 (5.2, 7.3)	5.3 (1.4)	5.4 (4.3, 6.3)
Attack duration (hours)	19.0 (10.2)	16.6 (12.6, 21.0)	16.4 (8.9)	14.9 (11.0, 21.0)
Allodynia	5.4 (3.7)	4.5 (2.0, 7.0)	5.1 (3.8)	4.0 (1.0, 8.0)
Perceived stress	18.0 (6.5)	17.5 (13.0, 22.0)	16.5 (6.6)	14.0 (10.0, 19.0)
Average daily moderate-to-vigorous physical activity (min)	40.1 (29.4)	32.3 (23.0, 49.6)	49.2 (37.9)	37.5 (24.6, 69.1)
Average daily caffeine intake (mg)	165.8 (190.2)	124.7 (43.1, 214.0)	122.7 (114.2)	104.8 (33.8, 185.1)
Average daily alcohol intake (g)	4.5 (11.3)	0 (0, 0.5)	3.6 (9.4)	0 (0, 0.2)
**Categorical Variables**				
	**Participants with Poor Sleep**		**Participants with Good Sleep**	
**Variable**	***n* (%)**		***n* (%)**	
Nausea frequency				
Low (0–33.3% of episodes)	32 (36.8%)		16 (41.0%)	
Medium (33.4–66.7% of episodes)	31 (35.6%)		11 (28.2%)	
High (66.8–100% of episodes)	24 (27.6%)		12 (30.8%)	
Photophobia (present at all episodes)	52 (59.8%)		25 (64.1%)	
Phonophobia (present at any episodes)	40 (46.0%)		13 (33.3%)	
Anxiety (GAD-7 ≥ 10)	27 (30.7%)		4 (10.3%)	
Depression (CES-D ≥ 16)	47 (53.4%)		9 (23.1%)	

Note. Percentages are for valid data. Abbreviations: PSQI = Pittsburgh Sleep Quality Index, BMI = body mass index, GAD = Generalized Anxiety Disorder Questionnaire, CES-D = Center for Epidemiological Studies-Depression, MMD = monthly migraine days, SD = standard deviation, 25% = 25th percentile, 75% = 75th percentile.

**Table 4:** Example hierarchical linear model results for migraine characteristics/clinical features predicting PSQI Global scores.

Step	Predictor	R^2^	ΔR^2^	F	b	95% CI
1		0.32	--	5.99 ***		
	Monthly migraine days				0.16 *	0.03, 0.29
	BMI				0.02	−0.08, 0.12
	Anxiety				0.75	−0.90, 2.40
	Depression				1.87 *	0.42, 3.32
	Perceived stress				0.12	<−0.01, 0.23
	Daily physical activity				−0.01	−0.03, 0.01
	Daily wear time				−0.01 *	−0.01, 0.00
	Daily caffeine intake				<0.01	<−0.01, <0.01
	Daily alcohol intake				0.01	−0.05, 0.06
2		0.32	0.01	5.46 ***		
	Monthly migraine days				0.15 *	0.02, 0.28
	BMI				0.02	−0.08, 0.12
	Anxiety				0.78	−0.88, 2.43
	Depression				1.87 *	0.42, 3.32
	Perceived stress				0.12	−0.01, 0.23
	Daily physical activity				−0.01	−0.03, 0.01
	Daily wear time				−0.01 *	−0.01, <0.01
	Daily caffeine intake				<0.01	<−0.01, <0.01
	Daily alcohol intake				0.01	−0.05, 0.06
	Monthly migraine days X BMI				0.01	−0.01, 0.03

Note. * *p* < 0.05, *** *p* < 0.001. Monthly migraine days and BMI were centered prior to computing the interaction term. Abbreviations: BMI = body mass index, PSQI = Pittsburgh Sleep Quality Index. Anxiety and depression were entered into the model as categorical variables. All other variables were continuous.

**Table 5:** Summary of migraine characteristics/clinical features in relation to overall and specific dimensions of sleep quality.

Migraine Characteristic or Clinical Feature	PSQI Sleep Quality Measure							
	Global Score		Sleep Efficiency		Sleep Quality		Daily Disturbance	
	b (95% CI)	*p*	b (95% CI)	*p*	b (95% CI)	*p*	b (95% CI)	*p*
Monthly migraine days	**0.16 (0.03, 0.29)**	**0.016**	**0.02 (<0.01, 0.03)**	**0.033**	<0.01 (<−0.01, 0.03)	0.289	0.03 (<−0.01, 0.06)	0.104
Pain intensity	0.32 (−0.06,0 69)	0.097	0.03 (−0.02, 0.07)	0.291	0.04 (−0.01, 0.08)	0.122	0.07 (−0.02, 0.17)	0.139
Attack duration ^a^	0.03 (−0.03, 0.09)	0.380	0.01 (<−0.01, 0.01)	0.112	<0.01 (<−0.01, 0.01)	0.739	<0.01 (−0.02, 0.02)	0.872
Allodynia	0.14 (−0.01, 0.29)	0.073	<0.01 (−0.02, 0.02)	0.997	0.02 (<−0.01, 0.04)	0.090	0.04 (<−0.03, 0.08)	0.069
Nausea frequency ^b^								
Medium	1.33 (−0.08, 2.75)	0.065	**0.22 (0.05, 0.39)**	**0.012**	0.08 (−0.11, 0.25)	0.413	0.04 (−0.34, 0.41)	0.843
High	0.80 (−0.66, 2.26)	0.278	0.06 (−0.11, 0.24)	0.475	0.06 (−0.13, 0.24)	0.553	0.16 (−0.23, 0.55)	0.424
Photophobia ^b^	−0.69 (−1.89, 0.50)	0.254	−0.09 (−0.24, 0.05)	0.214	−0.04 (−0.19, 0.11)	0.581	−0.10 (−0.41, 0.21)	0.541
Phonophobia ^b^	**1.85 (0.64, 3.07)**	**0.003**	**0.23 (0.09, 0.38)**	**0.002**	0.12 (−0.04, 0.27)	0.146	0.24 (−0.09, 0.56)	0.147

Note. Statistically significant findings are bolded. ^a^ Two outlier values were excluded (*n* = 125). ^b^ One participant was missing data (*n* = 126). Values displayed are unstandardized b values and 95% confidence intervals. Step 1 assessed the associations of the migraine characteristic/clinical feature of interest and BMI with the sleep outcome measure of interest, when controlling for potential confounders (depression, anxiety, stress, moderate-to-vigorous physical activity, caffeine intake, alcohol intake). The first step of all models was significant at *p* < 0.05. Inclusion of the interaction term in step 2 did not significantly improve prediction in any model. Sleep efficiency and sleep quality scores were transformed prior to analyses to achieve greater normality. Clinical features were either di- or trichotomized.

## Data Availability

The data presented in this study are available on request from the corresponding author.
